# Integrated cellular, proteomic and metabolomic profiling of immune-related adverse events in non-small cell lung cancer

**DOI:** 10.3389/fimmu.2026.1911262

**Published:** 2026-07-23

**Authors:** Natalie J. Smith, Bavani Gunasegaran, Anna McLean, Maija R. J. Kohonen-Corish, Alexandra Bucca, James Checkley, Jenny H. Lee, Jia (Jenny) Liu, Steven Kao, Michael Boyer, Kimberley Mander, Bea Brown, Xiao Suo Wang, John F O’Sullivan, Barbara Fazekas de St Groth, Helen M. McGuire

**Affiliations:** 1 School of Medical Sciences, Faculty of Medicine and Health, The University of Sydney, Sydney, NSW, Australia; 2 Program in Translational and Clinical Liver Cancer Research, Division of Medical Science, National Cancer Center Singapore, Singapore, Singapore; 3 Woolcock Institute of Medical Research, University of Sydney, Sydney, NSW, Australia; 4 Macquarie Medical School, Faculty of Medicine, Health and Human Sciences, Macquarie University, Sydney, NSW, Australia; 5 Sydney Local Health District, New South Wales Health, Sydney, NSW, Australia; 6 School of Medicine, Western Sydney University, Sydney, NSW, Australia; 7 Chris O’Brien Lifehouse, Sydney, NSW, Australia; 8 Faculty of Medicine and Health, University of Sydney, Sydney, NSW, Australia; 9 St Vincent’s Hospital, Sydney, NSW, Australia; 10 Garvan Institute of Medical, Research, Sydney, NSW, Australia; 11 NHMRC Clinical Trials Centre, Faculty of Medicine and Health, The University of Sydney, Sydney, NSW, Australia

**Keywords:** immune checkpoint inhibitor (ICI), immunophenotyping, irAE, mass cytometry, metabolomics, NSCLC, proteomics

## Abstract

**Introduction:**

Immune checkpoint inhibitor (ICI) therapy has become standard of care for late stage non-small cell lung cancer (NSCLC), producing durable responses in a subset of patients. However, inflammatory side eKects termed immune-related adverse events (irAEs) occur in up to 40% of ICI-treated NSCLC patients. Current approaches to alleviate irAEs include treatment with immune-suppressing corticosteroids. However, these treatments may undermine the eKicacy of ICIs by suppressing both the irAE and the anti-tumour immune response. To identify more specific therapeutic targets, a better understanding of the complex immunopathology underlying the development of irAEs in NSCLC is required.

**Methods:**

In this study, pre-treatment blood samples were prospectively collected from 72 NSCLC patients, including 23 who subsequently developed irAEs. Of these 72 samples, PBMCs from 59 were characterised using high-parameter mass cytometry. Plasma from 30 samples was analysed using the SomaScan platform that provides in depth characterisation of over 10,000 proteins, and the plasma metabolome of 30 samples was explored using liquid chromatograph-mass spectrometry (LC-MS).

**Results:**

A unique peripheral immunophenotype was observed in patients who subsequently developed irAEs, characterised by decreased memory B cell abundance, heightened Th2 immunity, and an increase in plasma cytokines. Investigation into baseline metabolites revealed dysregulation of fatty acid metabolism associated with development of irAEs. Analysis of additional paired PBMC (n = 17) and plasma (n = 12) samples collected early on treatment allowed exploration of the immunological, proteomic, and metabolic changes associated with irAE development. ICItreatment of patients who developed irAEs induced a significant increase in the abundance of CD8 memory cells and plasma histones. This points to the induction of a strong and potentially pathogenic immune response early following ICI treatment in patients who subsequently develop overt toxicity.

**Discussion:**

Overall, through application of a high-parameter multiomic approach, we have identified key cellular, proteomic and metabolomic features that predispose patients to developing immunotherapy toxicity. These findings provide insight into the complex biology underlying the development of ICI-related adverse events and inform potential treatment strategies.

## 1 Introduction

The introduction of immune checkpoint inhibitor (ICI) therapy to clinical practice has revolutionised the treatment landscape of non-small cell lung cancer (NSCLC), with durable responses seen in a subset of patients (1, 2). Checkpoint inhibitors are monoclonal antibodies that interfere with immune cell inhibitory pathways, such as the PD1/PDL1 axis, causing enhanced immune cell activation and tumour elimination (3). However, this enhanced immune activation can cause off-target inflammatory side-effects, termed immune-related adverse events (irAEs) (4). IrAEs develop in approximately 40% of NSCLC patients, most commonly affecting the cutaneous (dermatoses), endocrine (hypothyroidism), gastrointestinal (hepatitis, colitis), and pulmonary (pneumonitis) systems (5–7). Pneumonitis is the most serious irAE in NSCLC and accounts for 35% of irAE-related deaths (8, 9). Whilst ICI therapy may be continued with close monitoring following a mild irAE, severe events generally require suspension of ICI treatment and administration of immunosuppressants such as corticosteroids (10). Although broad immunosuppressants alleviate the symptoms of irAEs, they may also dampen anti-tumour immune activity. In fact, steroid use prior to ICI therapy commencement or within 2 months of treatment initiation is associated with reduced survival (11–13). Improved understanding of the immunopathology of irAE development is essential to uncover more targeted strategies to alleviate symptoms without undermining immunotherapy treatment efficacy.

The identification of targeted therapeutic strategies first requires understanding of the cellular and molecular processes that drive tissue damage at affected sites. Histological analysis of irAE skin lesions has revealed a mixed inflammatory infiltrate of T cells, eosinophils, and neutrophils (14–16). Similarly, infiltration of lymphocytes and neutrophils has been observed in colon and liver biopsies from patients experiencing ICI-related colitis or hepatitis (17, 18). As lung biopsies are not routinely obtained in the treatment of pulmonary irAEs, histopathological descriptions of ICI-related pneumonitis are rare. A comprehensive study of lung biopsies from 9 lung cancer patients who experienced ICI-related pneumonitis revealed elevated CD8 T cell infiltration, increased IFN-g production, and an activated humoral response in the lung (19). Furthermore, increased T cell abundance has been observed in bronchoalveolar lavage fluid (BALF) from patients experiencing ICI-related pneumonitis (20–22). Despite heterogenous presentations, an overarching feature of many irAEs is the infiltration of immune cells at the affected tissue site consistent with the notion that irAEs arise from dysregulation immune activation. However, why some individuals are pre-disposed to immune hyper-reactivity in response to checkpoint blockade remains unclear.

Peripheral blood offers a minimally invasive window into systemic immune cell dynamics and circulating soluble factors. High-dimensional single cell approaches have been widely applied to define the circulating immune phenotypes associated with irAE development in melanoma (23–25). Fewer studies have investigated immune phenotypes associated with irAE development in NSCLC, likely reflecting the later adoption of checkpoint immunotherapy to treat lung cancer. Single-cell sequencing of pre-treatment peripheral blood mononuclear cells (PBMCs) from 33 advanced NSCLC patients found heightened expression of inflammatory genes such as IL1B and CXCL2 in monocytes (26). Through application of single cell sequencing to pre-treatment T cells from 24 NSCLC patients, elevated CD4 Th2 cells were found to be associated with the development of pneumonitis (27). In-depth immunophenotyping of pre-treatment B cells from 46 NSCLC patients found that deficits in regulatory B cell abundance and IL-10 production were associated with irAE development (28). Furthermore, an early expansion of regulatory T cells following anti-PD1/L1 therapy was reported in 15 patients with NSCLC who experienced irAEs (29). Further in-depth exploration of immune cell phenotypes associated with irAE development in NSCLC is required to overcome the limitations of these published studies, which generally focused on only a restricted segment of the adaptive immune response.

Integration of blood immune cell profiling with measurement of the plasma proteome or metabolome allows characterisation of immune cells alongside the environment in which they circulate. To date, research into the plasma proteome in the context of irAE development has primarily included targeted analyses of cytokines or chemokines (29–32). Whilst such studies strengthen evidence for the involvement of known inflammatory mediators in the development of irAEs, they are limited to pre-selected targets. In contrast, untargeted proteomic approaches can identify novel proteins or pathways associated with immune activation and subsequent irAE development. Similarly, profiling of the plasma metabolome has provided insight into the physiological states associated irAE development in lung cancer (33). However, many studies have not integrated analysis of circulating soluble factors with detailed immune cell phenotyping. For example, elevated plasma concentrations of the CXCR3 ligands CXCL10 and CXCL11 have been associated with irAE development in NSCLC however corresponding cellular expression of CXCR3 was not assessed (29–32). One study of irAE development in 45 NSCLC patients integrated profiling of the plasma proteome and metabolome with immunological features, but immune cell profiling was limited to total B and T cell frequencies (34). Therefore, an integrated high-dimensional cellular, metabolomic, and proteomic profile of irAE development in late-stage NSCLC is yet to be established.

Accordingly, this study aimed to define the peripheral blood features associated with irAE development following anti-PD1/L1 treatment in late-stage NSCLC. By integrating high-dimensional immune cell profiling with plasma proteomic and metabolic analyses, we provide a comprehensive characterisation of the systemic changes associated with irAE development. We identify pre-treatment biological features that may predispose individuals to toxicity, including B cell dysregulation and alterations in immunoregulatory metabolic processes. Furthermore, paired analysis of pre- and on-treatment blood samples revealed evidence of widespread immune activation following checkpoint blockade in patients who subsequently developed adverse events. Collectively, these findings provide new insight into the cellular, proteomic, and metabolic pathways associated with irAE development in NSCLC. The immune and metabolic pathways identified here represent potential candidate therapeutic axes for future investigation. This may support the development of more targeted therapeutic strategies to prevent or treat irAEs while preserving anti-tumour immunity.

## 2 Methods

### 2.1 Sample collection and preparation

**Figure 1:**
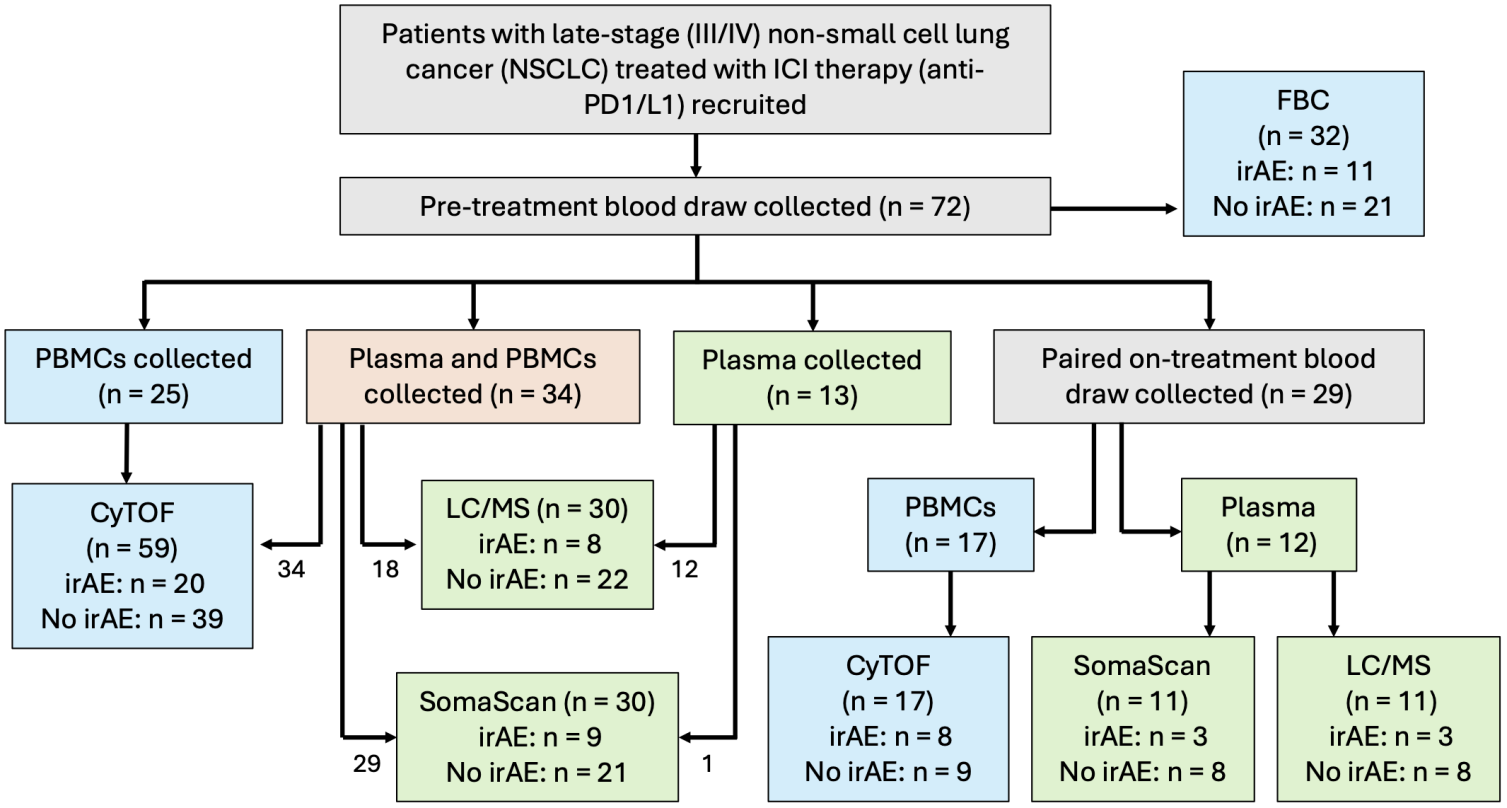
Study schema. Overview of the patient samples included in this study with summary of irAE status and analyses performed. Pre-treatment samples were collected from 73 ICI-treated NSCLC patients. Additional on-treatment blood samples were collected from 23 of these patients. Further details are provided in the Methods and in **Supplementary Tables 3**, **4**.

NSCLC patients were recruited from Royal Prince Alfred Hospital and Chris O’Brien Lifehouse. All samples were collected with informed written consent under ethics approval from the Sydney Local Health District Ethics Committee. Peripheral blood samples were collected prior to immunotherapy commencement from 72 patients and at 3 week intervals thereafter from 23 patients (**Figure 1**). For 32 patients, a pre-treatment full blood count (FBC) was recorded. Peripheral blood samples were collected into EDTA Vacutainer tubes (Becton Dickinson) and kept at room temperature (RT) for a maximum of 4 hours before processing.

For separation of plasma from blood cells, tubes were centrifuged at 800g for 15 minutes at RT. Plasma was removed and spun at 1600g for 10 minutes to deplete platelets. Platelet-depleted plasma was aliquoted into cryovials and stored at -80°C. Blood cells were diluted 1:1 in warmed PBS containing 5% heat-inactivated foetal calf serum (FCS)(PBS/FCS) before underlaying with 10mL of Ficoll-Paque™ PLUS density gradient media (Cytiva) and centrifuging at 500g at RT for 30 minutes with the brake disengaged. Peripheral blood mononuclear cells (PBMCs) were removed and washed twice in 50mL PBS/FCS, with centrifugation at 300g for 10 minutes at RT. Isolated PBMCs were then manually counted using a haemocytometer, with trypan blue to identify dead cells. Cells were cryopreserved in a CoolCell cryogenic freeze container (Corning) at -80°C, with each cryovial containing an aliquot of 2–4 million cells/mL in RPMI-1640 (Thermo Fisher Scientific) plus 10% dimethyl sulfoxide (DMSO) and 20% heat-inactivated FCS. Reference control PBMCs were prepared from one large blood draw (50mL) and frozen in 2 million cell aliquots to be stained for mass cytometric analysis with every batch of patient samples. After cryopreservation, PBMC samples were moved to vapour-phase liquid nitrogen for long-term storage.

### 2.2 Mass cytometry staining and acquisition

Pre-treatment (baseline) PBMC samples were analysed by mass cytometry from 59 patients, as previously described (35). Samples were stained in 4 batches with panels of 36–37 metal-tagged antibodies, as summarised in **Supplementary Table 1**. For 17 patients, paired on-treatment samples were analysed by mass cytometry using the Panel C reagent cocktail. Antibodies were either purchased in unlabelled format and conjugated in-house using the Maxpar X8 or MCP9 Antibody Labelling Kits (Standard BioTools) or purchased in labelled form from the Sydney Cytometry reagent bank. Cryopreserved PBMCs were rapidly thawed at 37°C in a water bath, then transferred into 10 mL of warm RPMI-1640 supplemented with 10% heat-inactivated FCS and 1:10000 Pierce Universal Nuclease (Thermo Fisher Scientific). Thawed samples were manually counted using a haemocytometer with trypan blue to identify dead cells. Live cell discrimination was performed with 1.25µM cisplatin. Cells were then stained with anti-CD45 antibodies for barcoding and PE (CCR10) or biotin (CD86) conjugated primaries (36). To facilitate standardisation across samples, 200000 cells from the barcoded reference sample were added to every sample before staining for the remaining surface antigens (37). Cells were fixed and permeabilised using the Foxp3/Transcription Factor Staining Buffer Set (eBioscience), then stained for intracellular targets. After staining, samples were fixed at 4°C overnight in 4% paraformaldehyde containing 1:4000 Cell-ID Intercalator-Ir (Standard BioTools). The following day, samples were spun down and resuspended in a solution of 90% FCS and 10% DMSO, then transferred to cryovials and frozen at -80 °C in a CoolCell container (Corning) until acquisition (38). On the day of acquisition, stained samples were thawed then washed in FACS buffer, Milli-Q water and then Maxpar Cell Acquisition Solution (CAS) (Standard BioTools). Samples were manually counted, resuspended at 0.8 x 106 cells/mL containing 1:10 EQ™ Four Element Calibration Beads (Standard BioTools), then passed through a 0.35µm nylon cell strainer snap-cap (Falcon). Acquisition was performed on a CyTOF™ 2-to-Helios™ upgraded mass cytometer with a flow rate of 30 µL/minute and event rate of up to 450 events/second.

### 2.3 Mass cytometry data analysis

Flow cytometry standard (.FCS) files were normalised in relation to the concurrently run EQ beads using the CyTOF Software. Normalised files were then imported into FlowJo 10.0.7 (BD Biosciences), and beads, cell doublets, dead cells, and neutrophils were removed by manual gating (**Supplementary Figure 1**). Samples were separated based on their CD45 barcode signal, then differentially barcoded samples and reference controls were exported for further analysis. Manual gating of mass cytometry files was performed in FlowJo 10.0.7 (BD Biosciences) as in **Supplementary Figure 2**, using the major cell subset marker definitions listed in **Supplementary Table 2**. Major cell populations containing less than 5000 events were excluded from downstream analysis in accordance with published recommendations that reliable estimation of population frequencies acquisition of adequate events (39). Cell type frequencies were defined as either a proportion of live PBMCs (% PBMCs) or as a proportion of their relevant parent population.

### 2.4 Plasma proteomics

The SomaScan Assay v5.0 profiles 10776 unique human proteins with approximately 11000 SOMAmer (Slow Off-rate Modified Aptamer) reagents in 130µL of plasma. Proteomic analysis of 30 plasma samples was performed by SomaLogic Inc. (Boulder, CO) according to standard protocol (40, 41). Briefly, SOMAmer reagents were synthesised with a fluorophore, photocleavable linker and biotin. These SOMAmers were incubated with diluted plasma samples (1:5, 1:200, and 1:20000), where they bound to target proteins. Bound proteins were then tagged with biotin, and unbound proteins were washed away. Non-specific complexes were disassociated and a polyanionic competitor was added to prevent rebinding. SOMAmers were then released from specific complexes by denaturing the proteins. Fluorophores were measured after hybridisation to complementary sequences on a microarray chip. Data quality control and standardisation steps were performed by SomaLogic Inc. including hybridisation normalisation, intraplate median signal normalisation, plate scaling, calibration, and adaptive normalisation to a reference control (41, 42). Normalised protein concentrations were reported in relative fluorescence units (RFU). Additional QC steps were performed in RStudio with the SomaDataIO package as described in Yang, Farias (43) (10.32614/CRAN.package.SomaDataIO). The limit of detection (LOD) was defined as the average expression level of each feature in concurrently run blank samples multiplied by 2 standard deviations. Features were excluded if greater than 15% of samples had a reading below the LOD. Additional features were excluded by low variance, defined as a coefficient of variation (CV) across the samples of less than 15%. After removal of non-human proteins and matching of SOMAmers sequence IDs to Universal Protein Resource (UniProt) IDs, 10193 proteins remained for downstream analysis.

### 2.5 Differential abundance analysis of proteomic data

Normalised and filtered RFU data was uploaded to ExpressAnalyst (https://www.expressanalyst.ca) and 10193 features were matched by UniProt ID annotation (44). Data was Log2 transformed and an additional filtering step was performed to remove low variance features by interquartile range (IQR) with a cutoff of 25%. To determine proteomic differences at baseline between the groups, Limma differential analysis was performed of the remaining 7948 features in ExpressAnalyst with correction for age and sex. Gene Set Enrichment Analysis (GSEA) was then performed with features ranked by fold change and mapped to KEGG pathways. FDR correction in ExpressAnalyst was performed with the Benjamini-Hochberg method. The normalised enrichment score (NES) was calculated to measure whether a pathway was overrepresented or underrepresented in patients with irAEs. The top 30 features from the cytokine-cytokine receptor pathway more abundant in the irAE group were imported into STRING to visualise interactions (https://string-db.org) (45). Experiments, databases, co-expression, neighbourhood, gene fusion, and co-occurrence were set as active interaction sources, with a medium confidence cut-off (0.4). A reference library containing all 7948 proteins included in differential analysis was uploaded as background for enrichment analysis. Disconnected nodes were hidden from the network and connections between nodes were selected to represent the strength of interaction between proteins. MCL clustering was performed with an inflation parameter of 3. These parameters were used for all STRING analyses unless otherwise stated. To determine changes in the proteome following immunotherapy treatment, protein expression in each patient was expressed as the on-ICI abundance divided by the paired pre-ICI abundance (fold change). Limma differential analysis was performed in ExpressAnalyst with correction for age and sex. With p < 0.05, 158 features were differentially abundant between the groups. Over-Representation Analysis (ORA) with features ranked by FC and mapped to KEGG pathways. These 84 proteins were imported into STRING to visualise connections between the proteins. Disconnected nodes were hidden from the network and connections between nodes were selected to represent the strength of interaction between proteins. MCL clustering was performed with an inflation parameter of 3.

### 2.6 Plasma metabolomics by LC-MS/MS

For targeted metabolomic analysis, a Triple quadrupole 6500 mass spectrometer (AB Sciex, Foster City, CA, USA) coupled with a Nexera LC-30AD UHPLC (Shimadzu Corporation, Kyoto, Japan) system used to detect hydrophilic metabolites in positive and negative ionization modes. Targeted metabolite profiling used in this study was established using reference standards for each individual metabolite to determine MS multiple reaction-monitoring (MRM) transitions as described previously (46). Authentic chemical standards were used to optimise individual MRM transitions, declustering potentials, collision energies and specific chromatographic retention times using a Waters HILIC column (Atlantis HILIC Sillica 3 um, 2.1 x 150 mm, Waters Corp., Milford, MA, USA). In brief, the chromatographic separation was achieved on a HILIC column using a gradient program with mobile A containing 0.1% formic acid, 10 mM ammonium formate in water; and mobile B containing 0.1% formic acid in 99.9% acetonitrile. The gradient was started at 95% B and maintained for 4 min before decreasing to 40% at 9 min, then maintained for 3 min before ramping back to 95% within one minute. The system was equilibrated at 95% for at least 10 more minutes before next injection. The flow rate was operated between 0.2 – 0.5 mL/min depending on elution requirements. The analysis software Sciex OS 3.1 (AB Sciex) was used for MRM Q1/Q3 peak integration of the raw data files. The peak area corresponds to the abundance of that metabolite, and the abundance values were then normalized to the pooled plasma extracts in the subsequent analysis to account for any temporal RT drift and/or MS variations in instrument performance. To determine changes in the metabolome following immunotherapy treatment, protein expression was expressed as the on-ICI sample divided by the paired pre-ICI sample (fold change) for each patient. Over-representation analysis (ORA) of significantly different metabolites was performed in MetaboAnalyst (https://metaboanalyst.ca) with the 91 quantified metabolites used as a reference metabolome and pathway identities from The Small Molecule Pathway Database (SMPDB) (47). Normalised metabolite abundance data was used for further statistical analyses and data visualisation.

### 2.7 Statistical analysis

GraphPad Prism version 11.0.0 was used for statistical tests and data visualisation. All statistical tests were two-sided unless stated otherwise. The Mann-Whitney test was used to assess statistical differences between two groups unless otherwise stated. A paired t-test was used to compare paired pre-treatment and on-treatment samples. The cut-off for significance for all tests was p < 0.05 unless otherwise stated. The two-stage step-up (Benjamini, Krieger, and Yekutieli) method was used to correct for multiple comparisons with FDR (Q) < 0.05 as cutoff for significance. Additional data visualisation was performed in MetaboAnalyst. Heatmaps were generated of z-score normalised features with Ward’s clustering. Partial Least Squares - Discriminant Analysis (PLS-DA) analysis was performed, with 2D scores plots displaying 95% confidence regions. PLS-DA features were assessed with variable importance in projection (VIP) score. Volcano plots were created in VolcaNoseR (https://huygens.science.uva.nl/VolcaNoseR/) (48).

## 3 Results

### 3.1 Patient characteristics

Pre-treatment blood samples were collected from 72 anti-PD1/L1 treated late-stage NSCLC patients and used to study the cellular, proteomic, and metabolomic features associated with subsequent irAE development. The study design, samples collected and analyses performed are summarised in **Figure 1**, **Supplementary Table 3**. The average age of patients was 69 and in the majority of patients (68%), the NSCLC was classified as adenocarcinoma (**Supplementary Table 4**). Pre-treatment samples were collected at a median of 45 days (range 0 – 218) before the start of anti-PD1/L1 therapy. Follow-up blood samples were taken from 29 patients (40%) at a median of 41 days (range 11 – 98) after the commencement of immunotherapy. Approximately a quarter of all patients (24%) received single agent immunotherapy, with the remaining patients receiving combination therapy with either chemotherapy alone (36%) or chemo-radiotherapy (ChemoRT) (40%). Immune-related adverse events (irAEs) occurred in 32% (23/72) of patients. Of the 23 patients who experienced irAEs, 13 (57%) had pneumonitis, 3 (13%) had hepatitis, and 3 (13%) experienced a cutaneous adverse event (**Supplementary Table 4**). The remaining 4 patients experienced an irAE affecting the endocrine, urinary, or gastrointestinal system. The median time to adverse event was 112 days (range 21 – 1027) days after commencement of anti-PD1/L1 therapy. The median follow-up time across all patients was 692 days (range 63 – 1750). There was no significant difference (p > 0.05) in either progression free survival (PFS) or overall survival (OS) between patients who did or did not experience irAEs (**Supplementary Figure 3**).

### 3.2 Cells involved in Th2 immunity are elevated in pre-treatment blood from patients who develop irAEs

**Figure 2:**
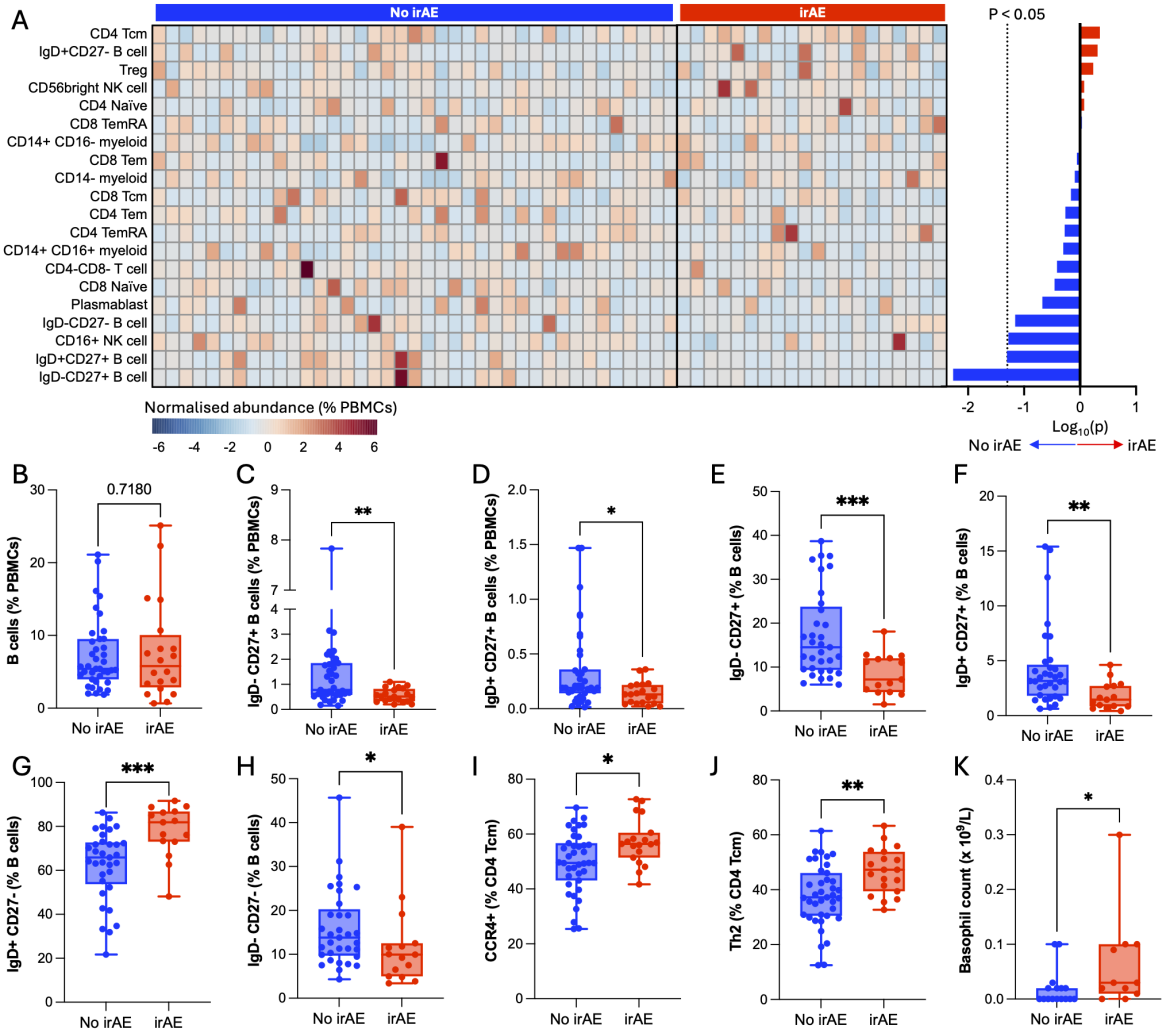
Mass cytometric analysis of pretreatment PBMCs to identify cellular correlates of subsequent irAE development. Pre-treatment peripheral blood mononuclear cells (PBMCs) from 59 anti-PD1/L1 treated NSCLC patients were analysed by high-parameter mass cytometry. **(A)** Heatmap of z-score normalised (mean-centred and divided by the standard deviation) abundance of 20 major cell populations identified by mass cytometry in 59 patients (**Supplementary Table 2**) grouped by future irAE status (no irAE n = 39, irAE n = 20). Significance determined by two-sided unpaired Mann-Whitney test and visualised as directional log_10_(p) values. Features ordered by ascending –log_10_(p) values with those more abundant in the irAE group in red and those less abundant in blue. **(B–J)** Box and whisker plots comparing subset proportions for the no irAE and irAE groups for **(B)** B cells (% PBMCs) **(C)** IgD^-^CD27^+^ B cells (% PBMCs), **(D)** IgD^+^CD27^-^ B cells (% PBMCs), **(E)** IgD^-^CD27^+^ (% B cells), **(F)** IgD^+^CD27^+^ (% B cells), **(G)** IgD^-^CD27^-^ (% B cells), **(H)** IgD^+^CD27^-^ (% B cells), **(I)** CCR4^+^ (% CD4 Tcm), and **(J)** CCR4^+^CXCR3^-^CCR6^-^ (Th2) (% CD4 Tcm). **(K)** A pre-treatment full blood count (FBC) was collected from 32/60 patients. Box and whisker plot shows basophil count (x 10^9^ cells/L). For all plots, the no irAE groups is shown in blue and the irAE group in red. Significance assessed by two-sided unpaired Mann-Whitney test. *P ≤ 0.05; **P ≤ 0.01; ***P ≤ 0.001.

We first aimed to explore the immune cell profiles that may pre-dispose certain patients to develop immune hyperreactivity following ICI therapy. To achieve this, pre-treatment PBMCs from 59 NSCLC patients were characterised by high-parameter mass cytometry (**Supplementary Figure 2**; **Supplementary Table 2**). Building on previous observations that autoantibodies are associated with irAEs, we first assessed associations between B cell phenotypes and irAE development (49–51). Whilst no significant difference in the abundance of total B cells was observed between the groups, CD27^+^ memory B cells (IgD^-^CD27^+^, p = 0.0024, and IgD^+^CD27^+^, p = 0.0151) were significantly less abundant in patients who developed irAEs (**Figures 2A–D**). Next, the distribution of B cell sub-populations was explored (**Figures 2E–H**). As the analysis of a small number of events introduces significant error to population frequency calculations, 11 samples (5 irAE, 6 no irAE) with fewer than 5000 B cell events were excluded from this downstream gating (39). Within the B cell compartment, IgD^-^CD27^+^ (p = 0.0007), IgD^+^CD27^+^ (p = 0.0016), and IgD^-^CD27^-^ (p = 0.0438) cells were significantly less abundant in patients who developed irAEs. A reciprocal increase in the abundance of naïve IgD^+^CD27^-^ cells (% B cells) was observed in patients who experienced adverse events (p = 0.0005). However, no association between naïve IgD^+^CD27^-^ B cell abundance (% PBMCs) and future irAE status was observed (**Figure 2A**). This could be explained by the positive correlation (p = 0.0073) observed between the abundance of B cells (% PBMCs) and the frequency of naive IgD^+^CD27^-^ cells (% B cells) (**Supplementary Figure 4**). As elevated plasma CXCL9, CXCL10, and CXCL11 have been associated with irAE development, the expression of CXCR3 on B cells was assessed (29–31). Whilst a trend towards heightened CXCR3 expression on IgD^-^CD27^+^ B cells was observed in the irAE group, this did not reach statistical significance (**Supplementary Figure 4**).

As collaboration between T and B cells is essential for a coordinated adaptive response, memory CD4 T cell phenotypes were next explored. Whilst we report no significant difference (p> 0.05) in the abundance of major T cell populations between the groups (**Figure 2A**), the expression of CCR4 on CD4 central memory (Tcm) cells was significantly higher in the irAE group (p = 0.0131; **Figure 2I**). This difference did not extend to total CD4 memory cells or CD4 effector memory (Tem) cells (**Supplementary Figure 4**). Given Th2 abundance has been associated with pneumonitis, co-expression of CCR4, CCR6, and/or CXCR3 was then applied to further classify CD4 T cells into Th1, Th2, and Th17 subsets (27, 52). In patients who subsequently developed irAEs, Th2 cells (CCR4^+^CXCR3^-^CCR6^-^) were significantly elevated within the CD4 Tcm compartment (p = 0.0032; **Figure 2J**), while there was no significant difference (p > 0.05) in the abundance of Th17 (CCR4^+^CXCR3^-^CCR6^+^) (**Supplementary Figure 4**). A trend towards lower Th1 (CCR4^-^CXCR3^+^CCR6^-^) abundance (% CD4 Tcm) was observed in patients who developed irAEs, but this did not reach statistical significance (p = 0.0557; **Supplementary Figure 4**). Given the role of eosinophils and basophils in Th2 immunity, their abundance was assessed in full blood counts (FBCs) collected from 32 patients. Basophils were significantly more abundant (p = 0.0461; **Figure 2K**) in pre-treatment blood from patients who subsequently developed irAEs. There was no significant difference in the abundance of eosinophils between the groups (p > 0.05; **Supplementary Figure 3**). Overall, analysis of pre-treatment immune cells revealed a deficit in memory B cells and elevated Th2-skewed immunity in peripheral blood from patients who subsequently experienced adverse events from checkpoint immunotherapy.

### 3.3 Patients who develop irAEs exhibit elevated inflammatory cytokines in pre-treatment plasma

**Figure 3:**
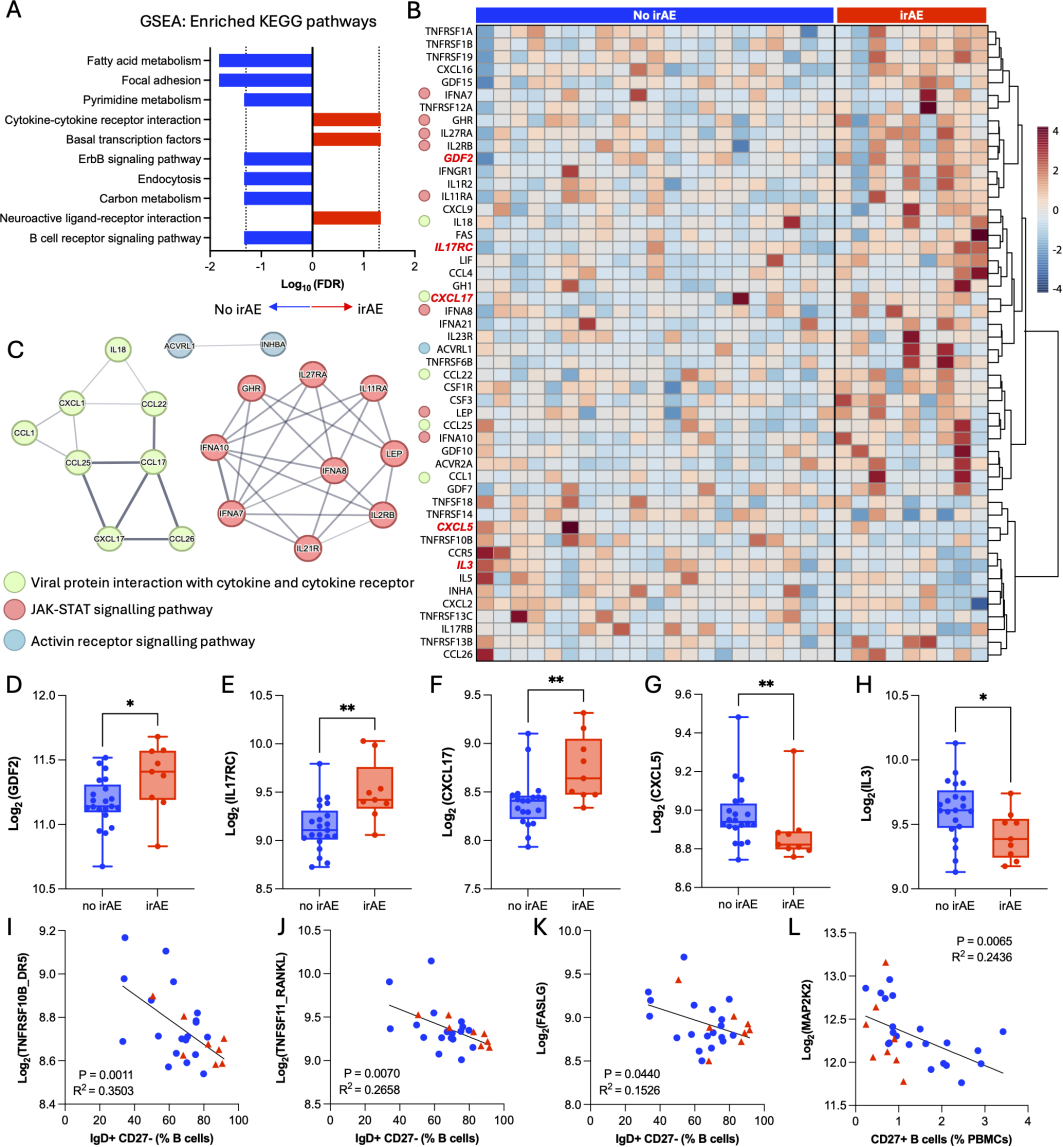
SomaScan analysis of pretreatment plasma to identify proteomic correlates of subsequent irAE development. Pre-treatment plasma samples from 30 ICI-treated NSCLC patients (no irAE n = 21, irAE n = 9) were analysed by the SomaScan 11K platform. **(A)** Limma differential analysis performed with correction for age and sex. Enriched pathways were explored by GSEA, with features ranked by fold-change (FC). Bar chart demonstrating the log10(FDR) value of the top 10 differentially abundant KEGG pathways. FDR values for pathways more abundant in patients without irAEs shown in blue. FDR values for pathways less abundant in patients with irAEs shown in red. **(B)** Heatmap of z-score normalised abundance of the top 50 differentially expressed proteins within the cytokine-cytokine receptor pathway with Ward’s clustering performed on features. Samples grouped by irAE status. Features annotated with STRING function enrichment pathway (green, viral protein interaction with cytokine and cytokine receptor; red, JAK-STST signalling pathway; Blue, activin receptor signalling pathway). Top 5 differentially abundant proteins plotted in **(D–H)** are indicated by red text. **(C)** Functional enrichments within the top cytokine-cytokine receptor proteins more abundant in patients who experienced irAEs visualised with STRING. MCL clustering performed and features annotated with their functional enrichment pathway. **(D–F)** Box and whisker plots demonstrating the abundance of **(D)** GDF2, **(E)** IL17RC, **(F)** CXCL17, **(G)** CXCL5, and **(H)** IL3 in patients with (red) and without (blue) irAEs. Significance determined by two-sided unpaired Mann-Whitney test. *P ≤ 0.05; **P ≤ 0.01. **(I-L)** Matched CyTOF (PBMC) and SomaScan (plasma) data from the same blood draw was available for 29 patients (no irAE n=21, irAE n=8). X-Y plots demonstrating the abundance of **(I-K)** IgD^+^CD27^-^ (% B cells; x-axis) plotted against **(I)** TNFRSF10B (DR5; y-axis), **(J)** TNFSF11 (RANKL; y-axis), and **(K)** FASLG (y-axis). **(L)** CD27^+^ B cells (% PBMCs; x-axis) plotted against MAP2K2 (y-axis). Simple linear regression performed with plots annotated with p and R^2^ values. No irAE group, blue circles; irAE group, red triangles.

As immune responses are mediated by soluble factors in circulation, we next investigated whether the immune cell phenotypes identified by mass cytometry were reflected in the plasma proteome. Accordingly, the SomaScan Platform was used to quantify over 10000 proteins in pre-treatment plasma samples from 30 NSCLC patients. First, an unbiased gene-set enrichment analysis (GSEA) was performed to identify the most differentially abundant KEGG protein pathways (**Supplementary Table 5**). This revealed that proteins involved in fatty acid metabolism (hsa01212; NES = -2.02; Q = 0.0150) and focal adhesion (hsa04510; NES = -1.74; Q = 0.01496) were significantly less abundant in pre-treatment plasma from patients who subsequently developed irAEs (**Figure 3A**; **Supplementary Table 6**). Given extensive evidence linking pre-treatment inflammatory cytokine abundance and irAE development, we next performed a targeted analysis of proteins involved in cytokine signalling (30, 32, 34). Notably, the cytokine-cytokine receptor interaction pathway (hsa04060) was the most significantly enriched in patients who developed adverse events (NES = 1.55; Q = 0.04626; **Figure 3B**). To further characterise interactions among proteins within this pathway, the top 30 proteins most abundant in the irAE group were analysed using STRING (**Supplementary Table 7**). This revealed three functional clusters enriched in the irAE group including JAK-STAT signalling, viral protein interaction with cytokine/cytokine receptor, and activin receptor signalling (**Figure 3C**). We next examined individual proteins within the cytokine-cytokine receptor pathway, identifying GDF2 (p = 0.0246), IL-17RC (p = 0.0017) and CXCL17 (p = 0.0020) as significantly enriched in the irAE group (**Figures 3D–F**). Conversely, CXCL5 (p = 0.0097) and IL-3 (p = 0.0279) were significantly less abundant in pre-treatment plasma from patients who developed adverse events. To integrate circulating immune cell and plasma proteomic data, matched mass cytometry, and SomaScan datasets from 29 patients were analysed. This revealed a significant inverse relationship between the frequency of IgD^+^CD27^-^ B cells (% B cells) and plasma levels of TNFRSF10B (DR5; p = 0.0011; **Figure 3I**), TNFSF11 (RANKL; p = 0.0070; **Figure 3J**), and FASLG abundance (p = 0.0440; **Figure 3K**). Proteins within the B cell receptor signalling pathway were also significantly reduced in patients who developed irAEs (has04662; NES = -1.728; Q = 0.04626; **Figure 3A**). To investigate whether reduced B cell activation may underlie the observed depletion of memory B cells in these patients, we next examined associations between proteins involved in B cell signalling and memory B cell abundance. Plasma MAP2K2, a key mediator of B cell receptor signalling, was significantly inversely correlated with CD27^+^ B cell frequency (% PBMCs; p = 0.0065; **Figure 3L**). Collectively, these findings demonstrate that irAE development is associated with alterations in fatty acid metabolism and circulating cytokine signalling proteins, together with changes in peripheral B cell-associated signalling pathways.

### 3.4 Circulating levels of fatty acid metabolism proteins are dysregulated at baseline in patients who experience irAEs

**Figure 4:**
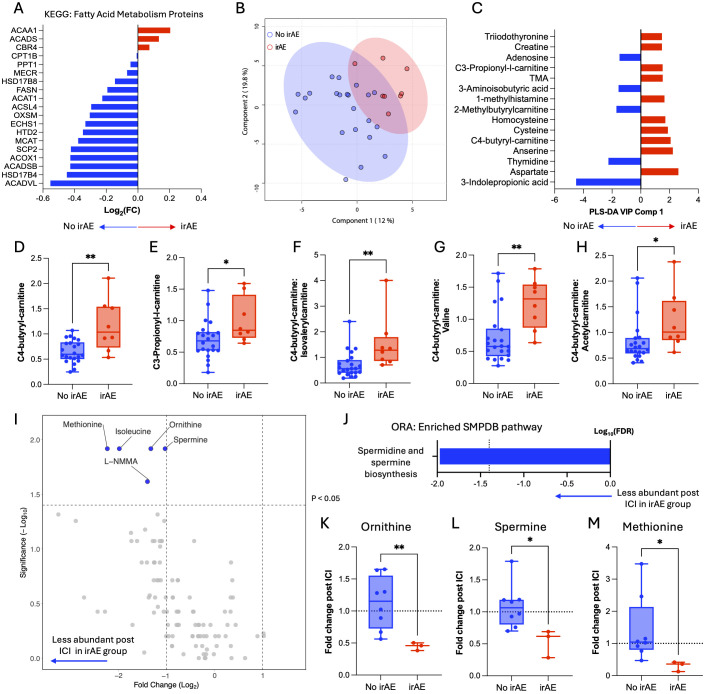
Metabolomic analysis of pretreatment and paired on-treatment plasma. **(A)** Limma differential analysis performed on plasma proteomic features from 30 ICI-treated NSCLC patients (no irAE n = 21, irAE n = 9) with correction for age and sex. Bar chart shows 19 proteins within the KEGG fatty acid metabolism pathway ranked by fold-change (FC). Proteins less abundant in patients with irAEs shown in blue. Proteins more abundant in patients with irAEs shown in red. **(B–H)** Pre-treatment plasma from 32 ICI-treated patients with NSCLC (no irAE n = 23, irAE n = 9) was analysed by LC-MS/MS, quantifying 91 metabolites. **(B)** Partial Least-Squares Discriminant Analysis (PLS-DA) analysis of baseline metabolites performed in MetaboAnalyst. Component 1 (x-axis) versus component 2 (y-axis) with the irAE group shown in red and no irAE group in blue. **(C)** Bar chart shows the top 15 features ranked by Variable Importance in the Projection (VIP) for PLS-DA component 1. VIP values less abundant in patients with irAEs shown in blue and those more abundant shown in red. **(D, E)** Box and whisker plots demonstrating the baseline abundance of **(D)** C4-butyryl-carnitine and **(E)** C3-propionyl-l-carnitine with significance determined by two-sided unpaired Mann-Whitney test. **(F–H)** Box and whisker plots demonstrating the ratio of C4-butyryl-carnitine to **(F)** isovalerylcarnitine, **(G)** valine and **(H)** acetylcarnitine with significance determined by two-sided unpaired Mann-Whitney test. **(I–M)** Exploration of the metabolomic changes induced by ICI therapy commencement in paired pre-treatment and on-treatment plasma samples from 11 NSCLC patients. The fold-change (FC) in metabolite abundance calculated as the abundance in the on sample divided by that in the pre sample. **(I)** Volcano plot demonstrating the 5 features less abundant on-ICI in patients from the irAE group with Log_2_ fold change on the x-axis and p-value from Mann-Whitney test on the y-axis. **(J)** Over-representation analysis (ORA) to explore Small Molecule Pathway Database (SMPDB) pathways enriched within the 5 differentially abundant features using the 91 quantified metabolites as reference metabolome. Bar chart demonstrating the log10(FDR) of the spermidine and spermine biosynthesis pathway. **(K–M)** Box and whisker plots demonstrating the FC in metabolite abundance for **(K)** methionine, **(L)** spermine and **(M)** ornithine. Significance determined by two-sided unpaired Welch’s t-test. For all plots, the no irAE groups is shown in blue and the irAE group in red. *P ≤ 0.05; **P ≤ 0.01.

**Figure 5:**
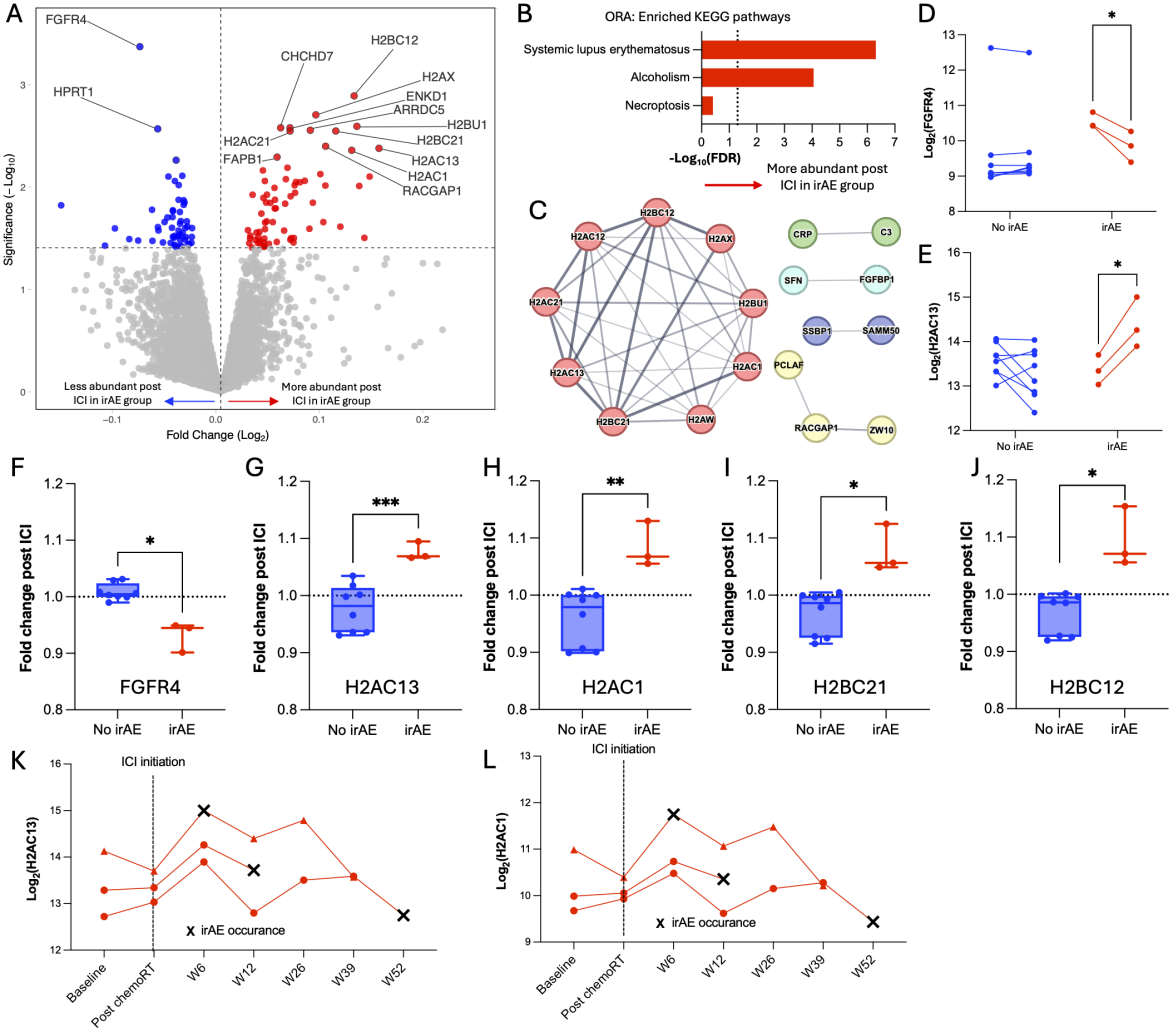
Proteomic changes induced by immunotherapy in patients who develop irAEs. Paired pre-treatment and on-treatment plasma samples from 11 NSCLC patients (no irAE n = 8, irAE n = 3) analysed by the SomaScan 11K platform. Fold change (FC) in protein abundance calculated by dividing on-therapy abundance by pre-therapy abundance for each protein. Limma differential analysis performed on protein FC values to identify proteins differentially abundant on-ICI in irAE group with correction for age and sex. **(A)** Volcano plot of Log_2_ fold change (x-axis) versus p-value (y-axis) with top 15 features ranked by p-value annotated. **(B)** Over-representation analysis (ORA) of the 158 differentially abundant features with p < 0.05 with features ranked by fold change and mapped to KEGG pathways. Bar chart demonstrating the –log10(FDR) of the significantly enriched KEGG pathways. Pathways enriched on-ICI in patients with irAEs shown in red. **(C)** Protein-protein networks within the 84 proteins more abundant on-ICI in the irAE group visualised with STRING. MCL clustering performed with features coloured by cluster. Disconnected nodes hidden. **(D, E)** Before-after plots showing the abundance of **(D)** FGFR4 and **(E)** H2AC13 in paired pre- and on-treatment samples with significance determined by paired t-tests using the two-stage step-up (Benjamini, Krieger and Yekutieli) multiple comparisons correction. **(F–J)** Box and whisker plots of the FC in abundance on-ICI of **(F)** FGFR4, **(G)** H2AC13, **(H)** H2AC1, **(I)** H2BC21, and **(J)** H2BC12 in patients with (red) and without (blue) irAEs. Significance determined by Welch’s t-test. **(K, L)** Chart demonstrating the abundance of **(K)** H2AC13 and **(L)** H2AC1 in longitudinal samples from 3 patients who experienced irAEs. Date of ICI commencement shown with a dashed line and timepoint of irAE occurrence indicated with black cross. *P ≤ 0.05; **P ≤ 0.01; ***P ≤ 0.001.

Given that proteins involved in fatty acid metabolism were less abundant in patients who experienced adverse events, we next sought to determine whether this was reflected at the metabolite level. To achieve this, pre-treatment plasma samples from 30 patients were analysed by LC-MS, and a targeted analysis of metabolites involved in fatty acid metabolism was performed (**Figures 4A–C**). Short-chain acylcarnitines including C4-butyryl-carnitine (p = 0.0061; **Figure 5D**) and C3-propionyl-l-carnitine (p = 0.0308; **Figure 5E**) were significantly elevated in pre-treatment plasma from the irAE group. As accumulation of short chain (C2-C5) acylcarnitines in blood is indicative of an impairment in mitochondrial beta-oxidation of fatty acids, these findings were consistent with the proteomic results (53).To further explore metabolomic changes at the pathway level, we examined metabolite ratios, which can provide a more robust representation of metabolic pathway activity. Ratios of C4-butyryl-carnitine to other fatty acid-related metabolites including isovalerylcarnitine (p = 0.0036), valine (p = 0.0043) and acetylcarnitine (p = 0.0156) were significantly higher in patients who experienced irAEs (**Figures 4F–H**). Collectively, these findings indicate dysregulation of fatty acid metabolism in pre-treatment plasma from patients who subsequently developed irAEs.

We next performed an untargeted analysis of metabolic changes induced by anti-PD1/L1 therapy. Five metabolites were identified as significantly less abundant (p < 0.05) on treatment in patients who developed adverse events (**Figure 4I**). Pathway over-representation analysis (ORA) revealed significant depletion of the spermidine and spermine biosynthesis pathway in the irAE group (Q = 0.0107) (**Figure 4J**). The individual differential metabolites within this pathway included methionine (p = 0.0175), spermine (p = 0.0192) and ornithine (p = 0.0021; **Figures 4K–M**). Of note, spermine is known to play an immunoregulatory role including suppression of pro-inflammatory cytokine synthesis and release (54, 55). Overall, patients who experienced irAEs were characterised by dysregulated fatty acid metabolism at baseline, together with a decrease in immunoregulatory metabolites in response to ICI therapy.

### 3.5 Plasma histone proteins increase in abundance following immunotherapy in patients who develop irAEs

To determine whether the metabolic alterations observed following immunotherapy were reflected at the protein level, we next analysed longitudinal changes in the plasma proteome. Paired pre- and on-treatment samples from 11 NSCLC patients were profiled using the SomaScan 11K Assay. Differential abundance analysis using Limma was performed to identify proteins that differed in on-treatment abundance between the groups (**Figure 5A**; **Supplementary Table 8**). ORA was then applied to the 158 differentially abundant proteins to identify significantly different KEGG pathways (**Supplementary Table 9**). Proteins within the systemic lupus erythematosus (SLE) KEGG pathway were significantly enriched following immunotherapy in patients who developed irAEs (hsa05322; Q < 0.0001; **Figures 5B–E**). At the individual protein level, fibroblast growth factor receptor 4 (FGFR4; 0.0272) decreased significantly in abundance following immunotherapy in patients who experienced irAEs. In contrast, multiple histone proteins including H2AC13 (p = 0.0003), H2AC1 (p = 0.0097), H2BC21 (p = 0.0238) and H2BC12 (p = 0.0367; **Figures 5F–J**) increased following immunotherapy in the irAE group. Profiling of additional longitudinal samples from 3 patients who experienced irAEs demonstrated the dynamic changes in plasma abundance of H2AC13 and H2AW (**Figures 5K, L**). As extracellular histones can promote inflammatory cytokine secretion, these findings are consistent with observed reduction in immunoregulatory metabolites following checkpoint blockade in patients who developed toxicity. Collectively, these results suggest that immunotherapy induces a shift towards a pro-inflammatory phenotype in patients who develop adverse events, characterised by increased circulating histones.

### 3.6 Immunotherapy induces changes in T and B cell abundance in patients who develop irAEs

**Figure 6:**
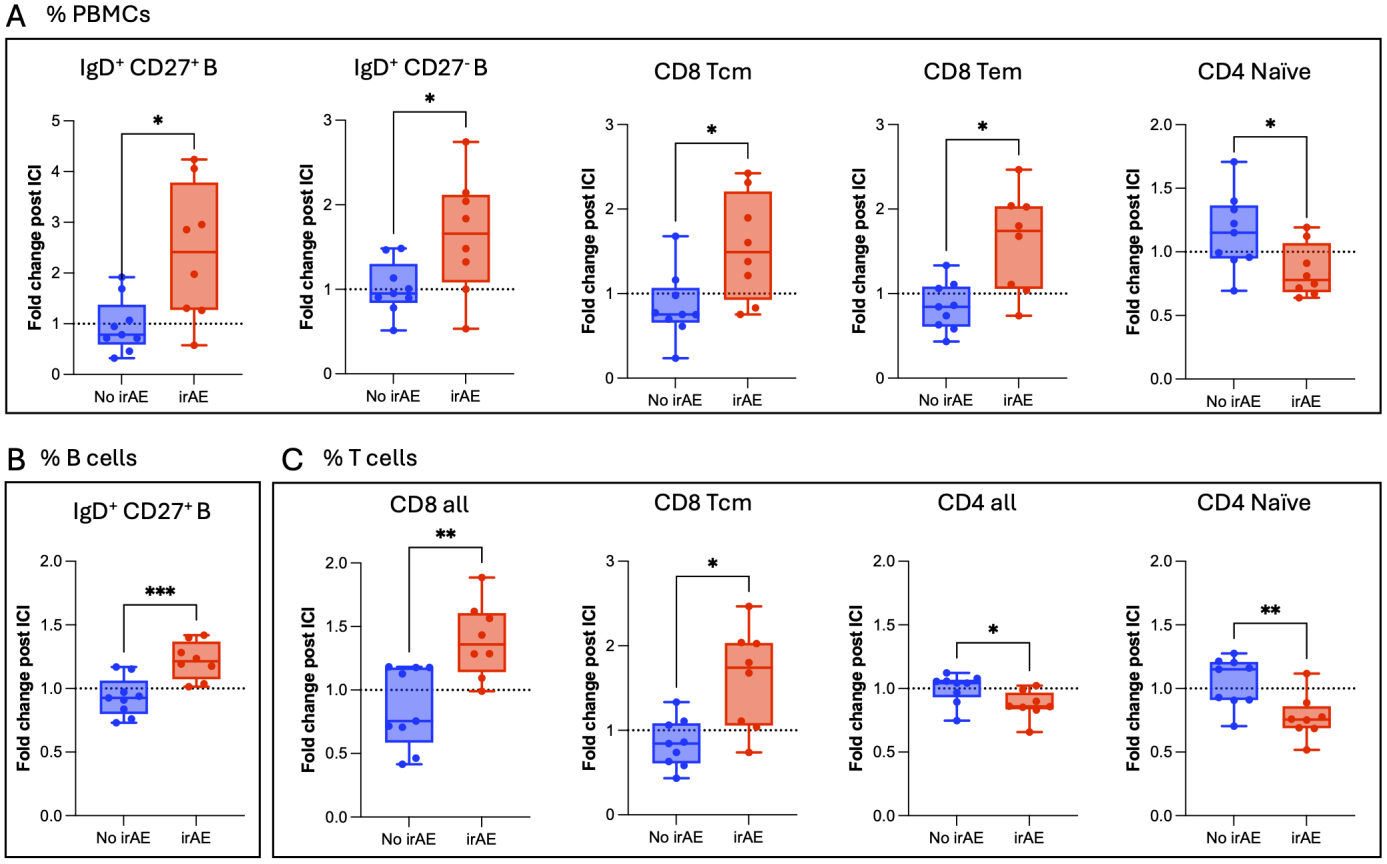
Analysis of cellular changes induced by immunotherapy in patients who develop irAEs. Paired pre-treatment and on-treatment PBMC samples from 17 NSCLC patients (no irAE n = 9, irAE n = 8) analysed by high-parameter mass cytometry. The fold change (FC) in immune cell abundance was calculated by dividing on-therapy abundance by pre-therapy abundance for each population. Box and whisker plots demonstrating the fold-change in immune cell abundance as a percentage of **(A)** PBMCs, **(B)** B cells, and **(C)** T cells. For all plots, the no irAE group is shown in blue and the irAE group in red. Significance assessed by two-sided unpaired Mann-Whitney test. *P ≤ 0.05; **P ≤ 0.01; ***P ≤ 0.001.

We next sought to determine whether the pro-inflammatory shift observed in on-therapy plasma in patients who developed irAEs was accompanied by corresponding alterations in immune cell phenotypes. Accordingly, paired pre- and on-treatment PBMC samples from 17 NSCLC patients were analysed by mass cytometry. For each immune population, the fold change in abundance following immunotherapy was calculated and compared between the groups. Several changes in immune cell abundance, expressed as a percentage of total live PBMCs (% PBMCs) were observed (**Figure 6A**). In the irAE group, IgD^+^CD27^+^ B cells (p = 0.0152), IgD^+^CD27^-^ B cells (p = 0.0464), CD8 Tcm (p =0.0152), and CD8 Tem (p = 0.0152) significantly increased following immunotherapy. In contrast, the proportion of naïve CD4 T cells (% PBMCs) significantly decreased on treatment in the irAE group (p = 0.0274). We next examined phenotypic changes within the B and T cell compartments (**Figures 6B, C**). IgD^+^CD27^+^ B cells increased significantly as a proportion of total B cells (p = 0.0055). CD8 T cells as a proportion of T cells increased (p = 0.0152), driven in part by an increase in CD8 Tcm cells (p = 0.0055). Conversely, total CD4 T cells (p = 0.0306) and naïve CD4 T cells (p = 0.0055) decreased as a proportion of total T cells within the irAE group. When expressed as a percentage of CD8 T cells, Tcm and Tem subsets did not differ significantly between the groups although total memory CD8 cells did increase significantly within the compartment (**Supplementary Figure 5**). This suggests that the observed changes in T cell subsets as a proportion of total T cells were primarily driven by a shift in the overall ratio of CD4 to CD8 cells. Overall, these findings are consistent with systemic immune activation following immunotherapy in patients who develop irAEs, aligning with the pro-inflammatory changes observed in the plasma proteome and metabolome.

## 4 Discussion

In this study, multiple high-parameter approaches were integrated to achieve a comprehensive assessment of the cellular, proteomic and metabolomic features associated with irAE development in NSCLC. Immune-related adverse events occurred in 32% (23/72) of patients, aligning with previous reports of irAE frequency in NSCLC (6, 56). Pneumonitis was the most frequently observed adverse event, occurring in 18% (13/72) of patients and accounting for 57% (13/23) of adverse events. While early clinical trials reported relatively low rates of ICI-related pneumonitis (1-5%), subsequent real-world studies have demonstrated a higher incidence (10-20%), in line with the frequency observed here (57–60). A weakness of the current study was the absence of toxicity grading for the documented irAEs, and future investigations should determine if the severity of irAEs correlates with the magnitude of the immune and metabolic changes documented here. Furthermore, the limited cohort size of this study prevented partitioning of patients by affected organ system or by treatment regimen (single agent or combined with chemo- and/or radiotherapy). Here, we report no significant difference in either progression-free or overall survival in patients who experienced irAEs. Previously reported associations between irAE development and survival outcomes differ by the severity of the adverse event experienced. Whilst mild or moderate irAEs have been associated with improved survival, patients who experience severe irAEs exhibit reduced survival either due to the irAE itself or the suspension of immunotherapy (6, 9, 61, 62). The lack of relationship between irAE development and survival outcomes reported here may therefore be due to mixed representation of patients with mild or severe adverse events in this cohort. However, as irAE development reflects systemic immune activation, future studies should assess whether the correlates of toxicity development defined here also associate with therapeutic response.

A distinct pre-treatment immune phenotype consisting of reduced CD27^+^ memory B cell populations was observed in peripheral blood from patients who subsequently developed irAEs. Peripheral B cell dysregulation has been widely associated with ICI-related toxicity, with previous studies linking deficits in IL-10^+^ regulatory B cells, an increased abundance of activated CD21^low^ B cells, and increased B cell clonality, with subsequent irAE development (28, 49, 50, 63–65). However, inconsistencies in the antigens used to classify B cells across studies complicates direct comparison of results. Here we used IgD and CD27 to categorise B cells as IgD^+^CD27^-^ (naïve), IgD^-^CD27^+^ (switched memory), IgD^+^CD27^+^ (non-switched memory), and IgD^-^CD27^-^ (double negative) populations (66). Although total B cell frequencies were similar between the groups, both switched (IgD^-^CD27^+^) and non-switched (IgD^+^CD27^+^) memory B cells were reduced at baseline in patients who developed irAEs. This phenotype mirrors that observed in autoimmune conditions such as systemic lupus erythematosus, lupus nephritis, and Sjögren’s Syndrome (67–69). Additionally, this B cell phenotype has been associated with the occurrence of interstitial lung disease in rheumatoid arthritis (70). Consistent with this, proteins involved in B cell receptor signalling were reduced in pre-treatment plasma, suggesting that B cell activation or memory differentiation is impaired in patients who develop irAEs. However, how memory B cell dysregulation is involved in the development of adverse events remains to be determined.

B cell subset distribution was skewed towards an abundance of naïve (IgD^+^CD27^-^) B cells in patients who developed irAEs. This is notable given evidence that naïve B cell repertoires can contain autoreactive clones that have escaped B cell tolerance in autoimmune disease (71, 72). The Fas/FASL axis is a key regulator of peripheral B cell tolerance, and disruption of this pathway has been implicated in autoimmunity (73). In line with this, an elevated abundance of naïve (IgD^+^CD27^-^) B cells was observed alongside reduced B cell expression of Fas in pre-treatment blood from melanoma patients who developed irAEs (50). In that study, a negative association between naïve B cell frequency and plasma FASL levels was observed, suggesting a potential impairment of apoptotic B cell regulation in patients who develop irAEs. Following immunotherapy, we observed significant B cell activation in patients who developed irAEs, as evidenced by an increase in expansion of memory B cell populations. Supporting this, humoral immune activation and increased IgG expression have been reported in lung tissue from patients from ICI-related pneumonitis (19). Additionally, deposits of complement and antibody have been observed in skin affected by cutaneous irAEs (14). Collectively, these data support a model in which pre-existing defects in B cell tolerance permit unrestrained B cell activation following checkpoint blockade therapy, contributing to irAE development.

The T cell phenotypes reported here further support a model of systemic immune dysregulation. While baseline CD4 and CD8 memory populations were not altered in patients who experienced adverse events, immunotherapy induced the expansion of CD8 Tcm and Tem subsets suggesting an induction of T cell activation. This aligns with previous studies linking CD4 memory T cell expansion and TCR diversity with the development of ICI-related toxicity in melanoma (24, 74). Increased CD8 infiltration has also been demonstrated in lung tissue or BALF from patients with ICI-related pneumonitis, suggesting trafficking of CD8 T cells to the tissue (19, 20, 75, 76). We did not assess cytokine secretion in the present study. However functional profiling of T cell cytokine secretion would be a valuable future direction to provide further mechanistic insight into T cell effector functions associated with irAE development. In addition, we report enrichment of Th2 CD4 memory subsets prior to immunotherapy in patients who experienced adverse events. This is in line with previous associations between elevated Th2 cells or plasma cytokines (IL-4, IL-13) and irAE development (27, 30, 77). Whilst we found no difference in the baseline abundance of IL-4 or IL-13, basophils which secrete IL-4 and IL-13 were found to be elevated in patients who developed irAEs (78). Whilst this is the first reported link between basophils and irAE development, elevated basophils in peripheral blood and tumour-draining lymph nodes have been associated with reduced survival following ICI therapy (79, 80). Consistent with associations between Th2 immunity and irAE development, elevated pre-treatment eosinophil counts have been associated with irAE development (81, 82). Additionally, eosinophils have been detected in affected tissue from patients experiencing gastrointestinal or cutaneous irAEs, and in BALF from patients with ICI-related pneumonitis (16, 17, 76). In fact, targeted inhibition of IL-5, a potent activator of eosinophils, was tested in a pilot study of 3 cancer patients with eosinophil-induced irAEs and was reported to result in long-term remission of symptoms (83). We found no association between pre-treatment eosinophil counts and irAE development, but this may in part have been due to our access to full blood counts from fewer than half the entire cohort of patients. Taken together, our study supports a link between Th2-skewed immunity which may drive the development of an allergic-like inflammatory immune response and the development of irAEs in NSCLC.

Despite previous reports associating Th17 signatures with irAE development, we found no expansion of Th17 CD4 T cells in patients who developed irAEs (25, 77). This discrepancy may have arisen due to the use of different methods of Th17 identification. Previous studies have identified Th17 cells by IL-17A or RORγt expression, whereas we used surface expression of chemokine receptors. However, we did find that IL-17RC protein was significantly more abundant in pre-therapy plasma from the irAE group. Evidence suggests that signalling through IL-17RC provides a negative feedback loop to Th17 cells that limits pathogenic cytokine production (84). Whilst few investigations into the source of soluble IL-17RC have been undertaken, increased detection in plasma is likely due to synthesis of a unique soluble form of the protein (85). Likewise, the role of soluble IL-17RC in inflammatory conditions is unclear. IL-17RC binds to both IL-17A and IL-17F, and administration of manufactured soluble IL-17RC has been shown to mediate Th17-driven inflammation (86). Accordingly, IL-17RC may be secreted as a regulatory mechanism to mediate the inflammatory immune environment already present at baseline in patients who develop irAEs. In fact, early studies have demonstrated that targeting of Th17 cytokines such as IL-17 and IL-6 may be a promising therapeutic approach to irAEs (25, 87, 88). However, as Th17 cells also play a role in the anti-tumour immune response, the effect of blocking this pathway on ICI treatment efficacy requires further investigation (89).

Given the central role of physiological factors in shaping the immune system, we sought to define the metabolic correlates of irAE development. Patients who developed irAEs exhibited an accumulation of short chain acylcarnitines and reduced abundance of fatty acid metabolism-related proteins in pre-treatment plasma. These findings are consistent with a previous study of irAE development in NSCLC and are indicative of impaired mitochondrial beta-oxidation of fatty acids (34, 53). Furthermore, elevated acylcarnitine levels due to mitochondrial dysfunction have been observed in synovial fluid from rheumatoid arthritis patients (90). As this metabolic dysfunction is known to promote inflammatory cytokine release, dysregulation in fatty acid metabolism may prime a pro-inflammatory immune state in patients who develop irAEs (91, 92). However, the relationship between plasma metabolic profiles and the metabolic regulation of specific cell types was not investigated here. In fact, a large source of variation in the concentration of plasma metabolites is the composition of the gut microbiome (93). Microbiome-derived metabolites such as short-chain fatty acids (SCFAs) can directly influence immune cell metabolism and inflammatory signalling (94). Specifically, SCFAs have been shown to induce an anti-inflammatory effect on T cells *in vitro* by reducing IL-17 production and increasing Treg abundance (95). Consistent with this, several studies have demonstrated associations between pre-treatment gut microbiota profiles, SCFA abundance, and the occurrence of severe adverse events following checkpoint immunotherapy (96–100). Faecal microbiota transplantation (FMT) from a healthy donor has been shown to mitigate ICI-related colitis and reduce CD8 T cell infiltration within the affected colon (101, 102). However, a variety of environmental factors including diet or antibiotic use can affect microbiome composition, making the generalisability microbiome profiles difficult across cohorts (103, 104).

Proteins involved in cytokine signalling were also elevated at baseline in patients who developed irAEs. Whilst increased concentrations of plasma chemokines have been widely associated with irAE development, our untargeted approach identified a novel association between plasma CXCL17 and irAE development. Notably, CXCL17 is not included in commonly available proteomic panels employed by previous studies of irAEs, which may explain why this relationship has not been previously identified (30, 32, 34). CXCL17 is constitutively expressed in tissues such as the lung and induces the migration of monocytes and immature dendritic cells (105). Whilst the cognate receptor for CXCL17 is yet to be defined, this chemokine is thought to facilitate migration of monocytes and dendritic cells from the vasculature to the tissue (106, 107). Increased CXCL17 expression has been detected in psoriatic skin specimens and in BALF from patients with interstitial pulmonary fibrosis (108, 109). In the context of irAE development, elevated plasma CXCL17 may be indicative of increased trafficking of antigen-presenting cells into the tissue, potentially enhancing subsequent adaptive immune system activation against the tissue after checkpoint blockade. However, this remains a speculative conclusion and further mechanistic studies are required to determine the functional role of CXCL17 in irAE pathogenesis.

Investigation of paired early on-treatment plasma samples revealed the induction of broad proteomic and metabolomic changes in plasma following immunotherapy. Metabolites involved in spermidine and spermine biosynthesis, including spermine itself, significantly decreased in abundance following ICI treatment in patients who developed irAEs. Given the known immunoregulatory role of polyamines such as spermine in suppressing pro-inflammatory cytokine production, this suggests a loss of metabolic restraint on immune cell activation following checkpoint blockade (54, 55). In parallel, increased circulating histone proteins were detected on treatment in patients who developed adverse events. As extracellular histones act as damage-associated molecular pattern (DAMP) proteins that promote inflammatory cytokine secretion, their elevation in consistent with heightened immune activation following therapy. Whilst this is the first study linking plasma histones and irAE development, dynamic increases in circulating histone concentrations have previously been reported after inflammatory insults, such as multiple trauma or sepsis (110–112). Consistent with a role for histones in development of irAEs, they have been shown in induce monocyte secretion of the CXCR3 ligands CXCL9 and CXCL10 (113), both of which increase in response to immunotherapy in patients who develop irAEs (29, 31). Strategies to neutralise the inflammatory action of histones are currently being considered for the treatment of systemic inflammation, autoimmunity, and sepsis (114, 115). As the on-treatment samples studied here were collected prior to the clinical onset of irAEs, the observed changes in plasma histone concentrations are unlikely to be a secondary consequence of irAE development. However, further mechanistic studies are required to determine how circulating histones contribute to irAE pathogenesis.

Our longitudinal plasma profiling indicated a deficiency in key immunoregulatory metabolites paired with an increased level of inflammatory plasma proteins following ICI therapy in patients who developed irAEs. However, the concentration of inflammatory factors in plasma is extremely variable, with plasma collected in the afternoon compared to the morning shown to contain higher concentrations of certain chemokines and cytokines (116). As investigations into the plasma proteome and metabolome were performed in a small cohort of patients, further study in a larger cohort is required to support the conclusions presented here. However, these findings collectively suggest a shift towards a pro-inflammatory systemic environment following immunotherapy in patients who develop adverse events. This is characterised by a reduced abundance of immunoregulatory metabolites and increased inflammatory protein release.

Collectively, we have defined a distinct peripheral immune phenotype present before anti-PD/L1 treatment in NSCLC patients who subsequently develop irAEs. Pre-existing B cell dysregulation and Th2-biased immunity may define a susceptible state, while metabolic impairments in fatty acid oxidation may further prime inflammatory signalling. Following blockade of PD1/L1, the observed expansion of effector immune populations and inflammatory mediators provide further evidence of altered immune activation in patients who develop irAEs. By integrating high-dimensional cellular, proteomic, and metabolic data, this study provides a comprehensive view of features associated with irAE development. However, these findings are exploratory and require validation in an independent cohort and as well as functional studies to determine the mechanisms through which the described cellular and molecular pathways influence irAE development. Future studies investigating these pathways may provide insight into strategies to mitigate off-target immune activation following immunotherapy, while preserving anti-cancer immune activity.

## Data Availability

The datasets presented in this study can be found in online repositories. The names of the repository/repositories and accession number(s) can be found in the article/**Supplementary Material**.
