# Supplementary material for: Integrated cellular, proteomic and metabolomic profiling of immune-related adverse events in non-small cell lung cancer
Source: Front Immunol. 2026 Jul 23;17:1911262. doi: 10.3389/fimmu.2026.1911262 (PMC13442671; doi:10.3389/fimmu.2026.1911262)
Supplement: Supplementary file 1 [file Table1.docx]

**Supplementary Figures**

**Supplementary Figure 1: Gating tree to de-barcode live CD45^+^ single cells.** Dot plot generated from FlowJo to demonstrate the gating hierarchy for data cleanup, with the headings of each plot indicating the population on which gates were drawn. EQ beads are removed in the first gate by selecting 140Ce^-^ DNA^+^ events. Single cells were then identified by event length and DNA signal. Next, dead cells and neutrophils were removed by selecting CD66b^-^ Cisplatin^-^ events. Samples were debarcoded accordingly to the metal-tagged CD45 antibody that they were stained with. Differentially barcoded samples are indicated in red text and were exported as separate files for downstream analysis.

**Supplementary Figure 2: Mass cytometry gating tree.** Dot plots generated in FlowJo from one representative PBMC sample. The plot headings (black text) identify the individual gated populations. Cell populations included in analysis are annotated with red text.

**Supplementary Figure 3: No association between PFS or OS and irAE occurrence.** Kaplan-Meier curve analysis of patients stratified by irAE status demonstrating **(A)** progression free survival (PFS) and **(B)** overall survival (OS). PFS and OS calculated from date of immunotherapy commencement. Plots annotated with median PFS and OS for each group. Significance assessed by Log Rank test, with p values annotated on plots.

**Supplementary Figure 4: Analysis of pre-treatment PBMCs by mass cytometry.** Pre-treatment peripheral blood mononuclear cells (PBMCs) from 51 ICI-treated NSCLC patients were analysed by high-parameter mass cytometry. **(A)** X-Y plot demonstrating the abundance of IgD^+^ CD27^-^ (% B cells; X axis) plotted B cells (% live; Y-axis). Simple linear regression performed with plots annotated with p and R^2^ values. The irAE group is plotted with red triangles and no irAE group with blue circles. **(B - G)** Box and whisker plots demonstrating **(B)** CXCR3^+^ (% IgD^-^CD27^+^ B cells), **(C)** CCR4^+^ (% CD4 memory), **(D)** CCR4^+^ (% CD4 Tem), **(E)** Th17 (% CD4 Tcm), **(F)** Th1 (% CD4 Tcm), and **(G)** Eosinophil count (x 10^9^/ L). For all plots, the no irAE group (n = 39) is shown in blue and the irAE group (n = 20) in red. Significance assessed by Kruskal-Wallis test.

**Supplementary Figure 5: Analysis of cellular changes induced by immunotherapy in patients who develop irAEs.** Paired pre-treatment and on-treatment PBMC samples from 17 NSCLC patients were analysed by high-parameter mass cytometry. The fold change (FC) in immune cell abundance was calculated by dividing post-therapy abundance by pre-therapy abundance for each population in paired samples. Box and whisker plots demonstrating the fold-change in immune cell abundance as a percentage of **(A)** live cells, **(B)** CD4 T cells, and **(C)** CD8 T cells plotted by future irAE status. For all plots, the no irAE group (n = 36) is shown in blue and the irAE group (n = 24) in red. Significance assessed by Kruskal-Wallis test. *P ≤ 0.05; **P ≤ 0.01; ***P ≤ 0.001.
